# Necroptosis-Related LncRNAs Signature and Subtypes for Predicting Prognosis and Revealing the Immune Microenvironment in Breast Cancer

**DOI:** 10.3389/fonc.2022.887318

**Published:** 2022-05-24

**Authors:** Yuhao Xu, Qinghui Zheng, Tao Zhou, Buyun Ye, Qiuran Xu, Xuli Meng

**Affiliations:** ^1^The Second Clinical Medical College, Zhejiang Chinese Medical University, Hangzhou, China; ^2^General Surgery, Cancer Center, Department of Breast Surgery, Zhejiang Provincial People’s Hospital (Affiliated People’s Hospital, Hangzhou Medical College), Hangzhou, China; ^3^Hangzhou Medical College, Hangzhou, China; ^4^Laboratory of Tumor Molecular Diagnosis and Individualized Medicine of Zhejiang Province, Zhejiang Provincial People’s Hospital (Affiliated People’s Hospital, Hangzhou Medical College), Hangzhou, China

**Keywords:** breast cancer, necroptosis, immune infiltration, immunotherapy, long non-coding RNAs

## Abstract

**Purpose:**

Necroptosis is a mode of programmed cell death that overcomes apoptotic resistance. We aimed to construct a steady necroptosis-related signature and identify subtypes for prognostic and immunotherapy sensitivity prediction.

**Methods:**

Necroptosis-related prognostic lncRNAs were selected by co-expression analysis, and were used to construct a linear stepwise regression model *via* univariate and multivariate Cox regression, along with least absolute shrinkage and selection operator (LASSO). Quantitative reverse transcription polymerase chain reaction (RT-PCR) was used to measure the gene expression levels of lncRNAs included in the model. Based on the riskScore calculated, we separated patients into high- and low-risk groups. Afterwards, we performed CIBERSORT and the single-sample gene set enrichment analysis (ssGSEA) method to explore immune infiltration status. Furthermore, we investigated the relationships between the signature and immune landscape, genomic integrity, clinical characteristics, drug sensitivity, and immunotherapy efficacy.

**Results:**

We constructed a robust necroptosis-related 22-lncRNA model, serving as an independent prognostic factor for breast cancer (BRCA). The low-risk group seemed to be the immune-activated type. Meanwhile, it showed that the higher the tumor mutation burden (TMB), the higher the riskScore. PD-L1-CTLA4 combined immunotherapy seemed to be a promising treatment strategy. Lastly, patients were assigned to 4 clusters to better discern the heterogeneity among patients.

**Conclusions:**

The necroptosis-related lncRNA signature and molecular clusters indicated superior predictive performance in prognosis and the immune microenvironment, which may also provide guidance to drug regimens for immunotherapy and provide novel insights into precision medicine.

## Introduction

Among the causes of global cancer incidence, breast cancer (BRCA) ranked the first in 2020 and was the fifth leading cause of cancer-related mortalities worldwide. According to the data reported, approximately 2.3 million new cases of BRCA were recorded in 2020 (1, 2). As a highly complex and heterogeneous disease with different molecular profiles, the decision-making of BRCA diagnostic and treatment were difficult, as well as the prediction of the clinical responses to therapeutic agents and prognoses (3). Thus, new effective targeted-therapeutic precision strategies are necessary.

The dynamic change in tumor microenvironment (TME) heterogeneity is considered to be the most important aspect of tumor heterogeneity, which depends on the tumor cells themselves in the microenvironment where the infiltrating immune cells and matrix together form an antitumor and/or pro-tumor network (4). The TME, a complex ecosystem composed of stromal cells, cancer cells, fibroblasts, chemokines, and immune cells (5), serves as a site of tumor cell growth and metastasis for promoting tumor immune escape, tumor growth, and metastasis (6–8), further influencing prognosis and prediction of response to specific treatments (9).

Necroptosis, seen as a novel form of programmed necrotic cell death, plays an important part in overcoming apoptosis resistance, triggering and amplifying antitumor immunity in cancer therapy (10, 11), similar to apoptosis in mechanism and necrosis in morphology (12).

LncRNAs are involved in regulating gene expression and transcription and post-transcription processes through chromatin modification (13) and then play an important role in dysregulation of gene expression and signaling pathways that are closely related to tumorigenesis, progression, and distant metastasis (14). According to recent research results, by participating in immune gene expression (TIM) and regulating inflammation, lncRNAs could influence the malignant phenotype of cancer by changing the tumor immune microenvironment (15–17).

Nevertheless, the prognostic value of necroptosis-related lncRNAs in BRCA has not been systematically demonstrated yet, and we still lack direct evidence about the predictive power of necroptosis-related genes (NRGs) in the prognosis and immunotherapy of BRCA. In this study, we identified a novel 22-prognostic-NRlncRNA signature and four NRLClusters to characterize the molecular features of BRCA using The Cancer Genome Atlas (TCGA) database. Subsequently, we further validated that the signature could serve as a robust independent predictor of prognosis and immuno-sensitivity response.

## Methods

### Data Acquisition and Processing

Primary expression data, corresponding clinical characteristics, and mutation data for 1,078 BRCA samples were extracted from the TCGA database. A total of 1,078 patients were assigned to train and test cohorts randomly with the ratio of 1:1 using the “createDataPartition” function in the “caret” package. Copy number variation (CNV) data were collected from the University of California, Santa Cruz (UCSC) website, and the immunology treatment response data were from The Cancer Immunome Atlas (TCIA) (18–20). Based on the expression files of 67 genes associated with necroptosis sorted out from the previous literature, 1,520 necroptosis-related lncRNAs were acquired by correlation analysis using the “cor” function in the “limma” R package (21).

### Construction and Validation of the Necroptosis-Related LncRNA Signature

Univariate Cox regression analysis was performed to screen out prognostic necroptosis-related lncRNAs in the train cohort using the “coxph” function in the “survival” R package. Then, the least absolute shrinkage and selection operator (LASSO) was used for the dimension reduction and K-fold cross-validation using the “cv.glmnet” function, which was multiplied by ten, and the optimal parameter was the λ value that corresponded to the lowest deviation. Subsequently, the riskScore of each patient was calculated based on each selected gene expression value multiplied by their coefficients, which were derived from the coefficient of multivariate Cox regression in the train cohort. The LASSO regression model was as follows:


$$\begin{array}{l} the\;riskScore\;formula\;=\;coefficients\;*expressing\;values\;of\;A\\ IncRNA+coefficients\;*\;expressing\;values\;of\;B\;IncRNA \end{array}$$

We divided patients into a high-risk and low-risk group with the median value of riskScore in the train cohort, and applied the value in the test and the entire cohort. The time-dependent receiver operating characteristic (ROC) curve using the “timeROC” R package and Kaplan–Meier (K-M) survival curves using the “survival” package were used to assess the signature’s predictive accuracy in the train, test, and entire cohort. Based on the value of riskScore, the K-M method was used to plot survival curves with log-rank *p*-value < 0.05 considered statistically significant. Moreover, we paid attention to the association between clinicopathological parameters and riskScore.

### Cell Culture

We purchased the normal breast epithelial cell line MCF-10A, and the epithelial BRCA cell lines MCF-7, T47D, MDA-MB-231, MDA-MB-468, and BT-549 from the American Type and Culture Collection (ATCC; Manassas, VA, USA). MDA-MB-231 and BT-549 cells were cultured in Dulbecco’s Modified Eagle’s Medium (DMEM) (ATCC; Manassas, VA, USA) supplemented with 10% fetal bovine serum (HyClone; Logan, UT, USA) and 1% antibiotic (100 IU/ml of penicillin and 100 µg/ml of streptomycin; HyClone; Logan, UT, USA). MCF-10A cells were cultured in DMEM/F12 medium supplemented with 20 ng/μl EGF, insulin, hydrocortisone, NEAA, 5% HS, and 1% P/S Solution (Procell; Wuhan, China). MCF-7 and MDA-MB-468 were cultured in Minimum Essential Medium (MEM) (Gibco BRL, USA) supplemented with 10% fetal bovine serum (HyClone; Logan, UT, USA) and 1% antibiotic (100 IU/ml of penicillin and 100 µg/ml of streptomycin; HyClone; Logan, UT, USA). T-47D cells were cultured in RPMI 1640 (HyClone, Logan, UT, USA) with 10% fetal bovine serum (HyClone; Logan, UT, USA). All the cell lines were incubated at 37°C, with a humidified atmosphere of 5% CO_2_.

### RNA Isolation and Real-Time PCR of LncRNAs in the Signature

Total RNAs were isolated from cells using the Trizol reagent (Invitrogen). The PrimeScript™ RT reagent Kit (Takara, Japan) was employed to perform reverse transcription to synthesize cDNA following the manufacturer’s protocol. Then, SYBR Green PCRMaster Mix (Applied TaKaRa, Otsu, Shiga, Japan) was used to conduct real-time PCR on Applied Biosystems 7500 Fast Real-Time RCR System (Applied Biosystems, Foster City, CA, USA). The primers of NRlncRNAs for qRT-PCR used in this research are shown as follows, which could also be seen in **Table 1**.

**Table 1:** The primers of NRlncRNAs for qRT-PCR used in this research.

Primer name	Primer sequence (5’ to 3’)
LINC00377 Forward	5′-GGAAAAGTGCATTTGCTTCGG-3′
LINC00377 Reverse	5′-TGACCTTGATGGCTTTTGATCC-3′
MEF2C-AS1 Forward	5′-ACTTGTTGCCTACTATCATACCTG-3′
MEF2C-AS1 Reverse	5′-ATAGCCATACAATAAGTTGCTCT-3′
LMNTD2-AS1 Forward	5′-AGTGACAGGCACTCACCTAC-3′
LMNTD2-AS1 Reverse	5′-TCTCCTGGAGCAGAGGGAATA-3′
LINC02446 Forward	5′-ATAGAGGCAAAGCAAGCCACT-3′
LINC02446 Reverse	5′-GTCACATCGTAGGAGGTGCTG-3′
GAPDH Forward	5′-CTGGGCTACACTGAGCACC-3′
GAPDH Reverse	5′-AAGTGGTCGTTGAGGGCAATG-3′

### Construction and Validation of a Prognostic Nomogram

To improve the accuracy of predicting the prognosis of BRCA patients, we further built a prognostic nomogram based on the NRL signature and other clinicopathologic features FOR forecast the 1-, 2-, and 3-year OS using the “rms” R package (22, 23), with corresponding calibration plots reflecting the predictive accuracy of the nomogram *via* the “calibrate” function (24).

### Prediction of Chemotherapy and Target Agent Response

*Via* the “pRRophetic” package, we calculated the half-maximum inhibitory concentration (IC_50_) to evaluate the difference in drug response between different groups in the Genomics of Drug Sensitivity in Cancer (GDSC) database (25, 26) using Ridge’s regression, along with 10-fold cross-validation for the purpose of improving the accuracy of the prediction (27, 28).

### Assessment of Immune Cell Infiltration, Immune Checkpoint, and Immunotherapy

We calculated immune cell infiltration in each sample using the single-sample gene set enrichment analysis (ssGSEA) algorithm and found out the significantly differential pathways between the two groups with gene set variation analysis, using the “GSEABase” and “GSVA” packages. Furthermore, we further performed CIBERSORT algorithm, which used expression data to assess the stromal and immune cells using the “e1071”, “parallel”, and “preprocessCore” packages, and Spearman rank correlation analysis for analyzing the correlation between the riskScore and the relative expression level of the 22 tumor-infiltrating leukocytes using the “limma” package. We also probed into the expression levels of known immune checkpoint genes in high- and low-risk groups. Furthermore, we discussed the relationship between the NRLscore and clinical PD-L1 and CTLA-4 subtypes in the TCIA database using Student’s *t*-test.

### Tumor Mutation Burden Analyses

Using mutation data of BRCA patients, the waterfall plot was generated with Maftools R-package to compare the differences in gene mutation frequency between high- and low-risk BRCA patients. We analyzed the correlation between TMB and riskScore using Student’s *t*-test and Spearman rank correlation analysis.

### Profiles of Necroptosis-Related LncRNAs Identified Four Distinct Molecular Phenotypes of BRCA

By using hierarchical agglomerative clustering on the basis of Euclidean distance and Ward’s linkage (29), BRCAs with qualitatively varying necroptosis-related lncRNA expressions were clustered. The K-means method was applied to classify patients for further study using the “ConsensusClusterPlus” package (30).

### Statistical Analysis

All statistical data analyses were performed in GraphPad Prism (version 7) and R software (version 4.1.1). Wilcoxon test (for comparison between two groups) and Kruskal–Wallis test (for comparison among more than two groups) were applied to discuss the statistically significant differences. Log-rank test was used to determine the differences in overall survival between different risk groups and molecular clusters. The correlations between the two were evaluated *via* Spearman’s correlation analysis. *p* < 0.05 was considered to be the threshold for statistical significance, and all the *p*-values mentioned in the paper were two-tailed.

## Results

### Genetic Variation Landscape of Necroptosis-Related Genes in BRCA

**Figure 1:**
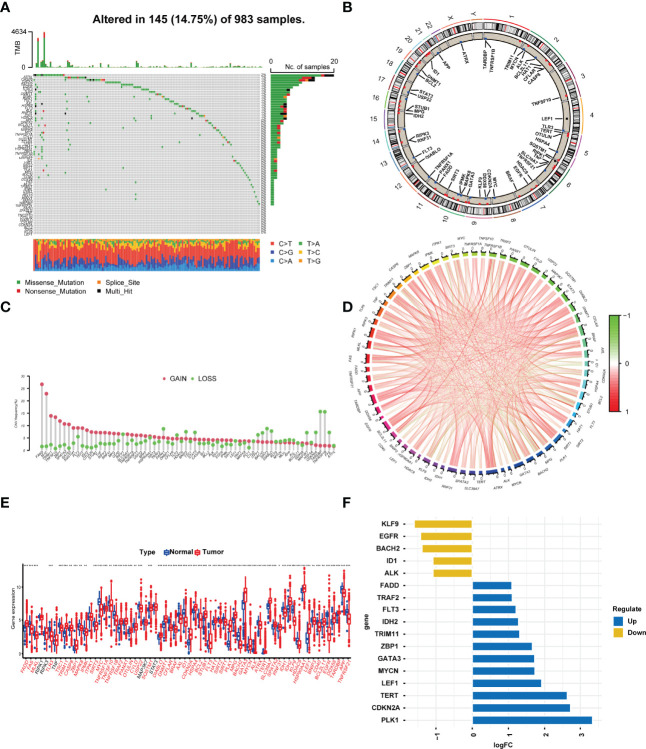
Profile of the 67 necroptosis-related genes in BRCA. **(A)** In all, 145 of 983 (14.75%) BC patients experienced 67 necroptosis-related gene alterations. **(B)** The location of the 67 necroptosis-related genes in chromosomes. Blue points represented that the gene mainly had CNV deletion, red points represented that the gene mainly had CNV amplification. **(C)** CNV mutation frequency of the 67 necroptosis-related genes. This column represents the frequency of change. Deletion frequency is represented by green dots, while amplification frequency is represented by pink dots. **(D)** Expression interaction of the 67 necroptosis-related genes in BRCA. The lines connecting the necroptosis-related genes show how they are correlated with each other, with positive associations in red and negative associations in green. **(E)** Expression of the necroptosis-related genes in normal tissues and BRCA tissues. Genes with red color represented the differentially expressed genes. **(F)**. The value of logFC of the genes. *P<0.05; **P<0.01; ***P<0.001.

According to the previous literature, we confirmed 67 NRGs (22). Genomic mutations were common in these genes, with genetic changes occurring in 145 (14.75%) of 983 patients, in which ATRX (2%) had the most genetic alteration, and a mutation frequency of 1% was observed in CASP8, GATA3, BACH2, EGFR, STAT3, TLR3, DNMT1, BRAF, RIPK1, TSC1, AXL, HSPA4, FLT3, ALK, RNF31, and IDH1 (**Figure 1A**). The location of the 67 NRGs in human chromosomes can be seen in **Figure 1B**. Meanwhile, we found that most genetic variations had CNV amplification (**Figure 1C**). In order to discover the interaction of the 67 NRGs directly, we constructed a network to show the connection between each other (**Figure 1D**). The differential analysis in normal breast tissue and tumor tissue revealed that, except for RIPK1, RIPK3, TNF, MAP3K7, and STAT3, all the other genes showed significantly differential expression in BRCA (**Figure 1E**). PLK1, CDKN2A, TERT, LEF1, MYCN, GATA3, ZBP1, TRIM11, IDH2, FLT3, TRAF2, and FADD were seen as upregulated genes in BRCA, with the value of log FC of TERT being the highest (log FC = 2.627). ALK, ID1, BACH2, EGFR, and KLF9 were seen as downregulated genes in BRCA, with the value of log FC of KLF9 being the highest (log FC = −1.601) (**Figure 1F**). The alteration and genetic variation of NRGs acted as an important part in regulating the happening, aggravation, and prognosis of BRCA.

### Identification of Prognostic Necroptosis-Related LncRNAs

**Figure 2:**
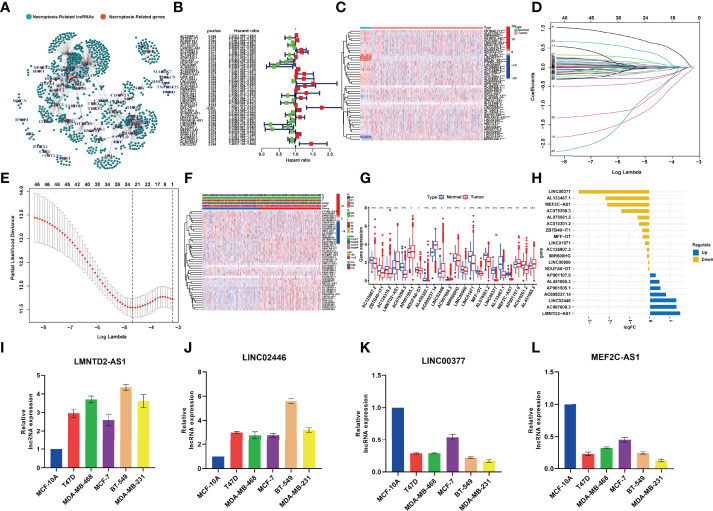
**(A)** The network between necroptosis-related genes and lncRNAs (correlation coeffcients >0.4 and *p* < 0.001). **(B)** The prognostic necroptosis-related lncRNAs extracted by univariate Cox regression analysis. **(C)**. The heatmap that showed the expression profiles of 46 prognostic lncRNAs, which showed significant difference between normal and cancer tissues. **(D)** The 10-fold cross-validation for variable selection in the LASSO model. **(E)** The LASSO coeffcient profile of 22 necroptosis-related lncRNAs. **(F)** Correlation analysis between risk groups and clinical features. **(G)** Expression of the 22 NRlncRNAs in the model between normal tissues and BRCA tissues. **(H)** The value of logFC of the 22 NRlncRNAs. **(I–L)** qRT-PCR results showing the expression of lncRNAs in the normal breast and five breast cancer cell lines. *P<0.05; **P<0.01; ***P<0.001.

The clinical data and transcriptome data were retrieved from the TCGA database, including 1,096 BRCA specimens and 112 normal specimens. We performed Spearman correlation analysis between the lncRNAs and NRGs, and 1,520 necroptosis-related lncRNAs (NRlncRNAs) were sorted out with the filter criteria of correlation coefficients >0.4 and *p* < 0.001 (21, 22, 31, 32) (**Table S1** and **Figure 2A**). Forty-six prognostic NRlncRNAs in BRCA were extracted by univariate analysis, of which the significant filtering condition was *p* < 0.05 (**Table S2** and **Figures 2B, C**).

### Establishment and Validation of Prognostic Signature for NRlncRNAs in BRCA

LASSO‐penalized Cox regression was used to establish the following equation based on the expression of 22 NRlncRNAs in the train cohort (**Figures 2D, E**):


riskScore=[(0.0314*AC125807.2 )+(0.3155*ZBTB40−IT1) +(−0.0010*AC124319.2)  +(−0.0086*LMNTD2AS1)+(0.0394*AC079298.3) +(0.0044*AP001505.1) +(−0.2655*NDUFA6−DT)+(−0.9949*AL450322.1)+(0.0230*AC009237.14)+ (−0.0061*LINC02446) +(0.3555*AC007608.3)+(0.0979*MIR600HG) +(0.1248*LINC00900) +(−0.0385*LINC01871) +(0.6195*MFF−DT)+ (0.0054*AL078581.2) + (−0.7159*LINC00377)+(−0.0019*AL133467.1)+(−0.6298*MEF2C−AS1)+(−0.1186*AP001107.9)+(−0.0838*AC010201.2)+(−0.1161*AL451085.3)

Compared with the normal breast tissue, the expression levels of LMNTD2-AS1, AC007608.3, and LINC02446 were significantly higher in the BRCA group, while the expression levels of LINC00377, AL450322.1, MEF2C-AS1, and AC079298.3 were lower with the filter criteria of *p* < 0.05 and |log FC| > 1 (**Figures 2F–H**). qRT-PCR analyses verified the results of bioinformatics analysis, revealing that expression levels of LncRNA LINC00377 MEF2C-AS1 were significantly downregulated in BRCA cell lines compared with the normal breast cell line (*p* < 0.05, **Figures 2I–L**).

**Figure 3:**
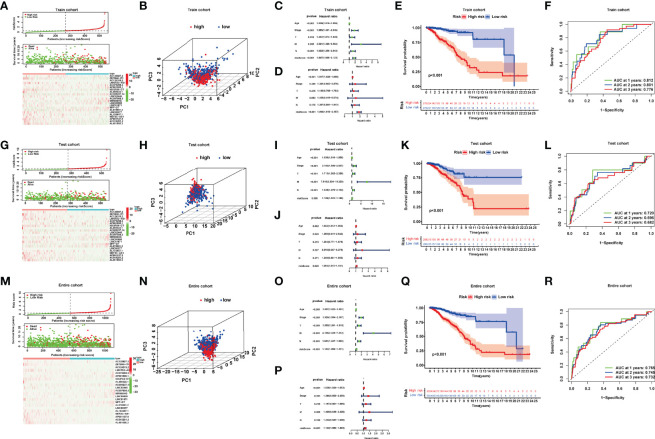
**(A, G, M)** Risk curve; scatter plot of vital status by risk score; heatmap of the 22 lncRNAs’ expression in the train, test, and entire cohort. **(B, H, N)** Principal component analysis (PCA) of BRCAs based on the riskScore in the train, test, and entire cohort. **(C, D, I, J, O, P)** Univariate and multivariate Cox regression analyses in the train, test, and entire cohort. **(E, K, Q)** Kaplan–Meier curves of the high- and low-risk patients in the train, test, and entire cohort. **(F, L, R)** Time-dependent ROC curves for predicting 1-, 2-, and 3-year OS in the train, test, and entire cohort.

Combining the coefficients and expression of the above NRlncRNAs, we computed the riskScore for each BRCA patient. The entire cohort included 1,078 samples, which were randomly divided into a train cohort (540 samples) (**Table S3**, Sheet train) and a test cohort (538 samples) (**Table S3**, Sheet test) according to the ratio of 1:1. Based on the value of the median riskScore in the train cohort, we divided patients into high- and low-risk groups in the train and test cohort, respectively. A total of 270 patients were categorized into the high-risk group, and 270 patients were categorized into the low-risk group in the train cohort while 258 patients were categorized into the high-risk group and 280 patients were categorized into the low-risk group in the test cohort (**Figures 3A, G, M**).

We performed principal component analyses (PCAs), and the result indicated good discriminative performance of the NRlncRNA model in the train, test, and entire cohort (**Figures 3B, H, N**). The K-M survival curve displayed that compared with the high-risk group, the OS of patients in the low-risk group was significantly longer*p* < 0.001 in three cohorts) (**Figures 3E, K, Q**). The area under curve (AUC) values for the 1-year (0.812), 2-year (0.801), and 3-year (0.776) survival rates in the train cohort, 1-year (0.720), 2-year (0.696), and 3-year (0.682) survival rates in the test cohort, and 1-year (0.765), 2-year (0.745), and 3-year (0.732) survival rates in the entire cohort showed favorable specificity and sensitivity of the signature in predicting OS (**Figures 3F, L, R**). Lastly, we performed univariate and multivariate Cox regression analyses internally and externally, implying that age and riskScore, which served as high-risk factors, were significantly correlated with OS (*p* < 0.05, HR > 1) (**Figures 3C, D, I, J, O, P**). These results revealed that the NRlncRNA signature could efficiently and independently identify the risk of BRCA prognosis.

### Stratified Prognostic Analysis and Association of NRlncRNA Signature With Clinical Logical Features

**Figure 4:**
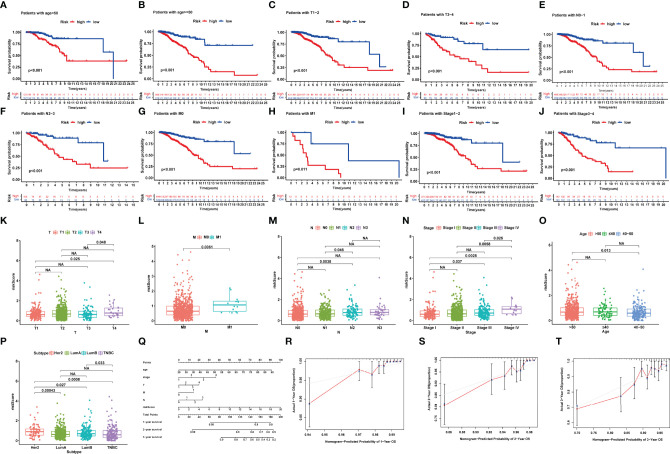
**(A–J)** Kaplan–Meier survival analysis for BRCA patients with diverse clinical characteristics of **(A, B)** age, **(C, D)** T-Stage, **(E, F)** N-stage, **(G, H)** M-stage, and **(I, J)** AJCC stage. **(K–P)** Correlation between signature and clinical characteristics. **(Q)** The nomogram plot integrating NRlncRNA riskScore, age, T-, N-, and M-classification. **(R–T)** The calibration plot for the probability of 1-, 2-, and 3-year OS. NA, P>0.05.

To further demonstrate the predictive power of the prognostic model, we performed the K-M analysis by log-rank test for the purpose of assessing the prediction capacity of multiple clinical characteristics on BRCA patients after stratifying the patients into subgroups of age (≥55 and <55), AJCC stage (I + II and III + IV), T stage (T1-2 and T3-4), N stage (N0-1 and N2-3), and M stage (M0 and M1). The results revealed that the NRlncRNA signature had good prognostic ability in each clinical subgroup (**Figures 4A–J**).

We also focused our attention to the association of NRlncRNA signature with age, T stage, M stage, N stage, and AJCC stage in BRCA patients. The riskScore was significantly higher in the T4, M1, and stage IV groups compared with the other corresponding groups (**Figures 4K–O**). However, we also observed that HER-2-positive BRCA indicated a higher riskScore, while Luminal BRCA indicated a lower riskScore (**Figure 4P**).

### Development of the Nomogram for Prognostic Prediction

To further enhance the prognostic prediction power, we developed a nomogram that integrated age, TNM stage, and riskScore (**Figure 4P**). Then, we built calibration curves, of which the *y*- and *x*-axis represent the actual and predicted survival rate from the nomogram to assess the predictive accuracy and clinical practicability of this nomogram. The calibration plot for OS probability at 1, 2, and 3 years suggested satisfactory consistency between the actual and predicted survival probabilities (**Figures 4Q–T**).

### Prediction of Chemotherapy or Target Agent Response

**Figure 5:**
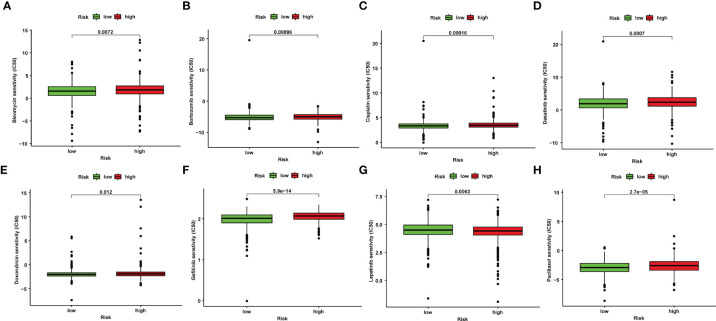
Correlation between NRlncRNA signature and drug sensitivity. Box plots for the estimated IC_50_ of drugs between high- and low-risk BRCA patients. Bleomycin **(A)**, Bortezomib **(B)**, Cisplatin **(C)**, Dasatinib **(D)**, Doxorubicin **(E)**, Gefitinib **(F)**, Lapatinib **(G)**, Paclitaxel **(H)**.

The IC_50_ values of several chemotherapeutic agents were used to evaluate chemotherapeutic response to BRCA patients. We observed that low-risk patients had dramatically reduced IC_50_ values of bleomycin, bortezomib, cisplatin, dasatinib, doxorubicin, gefitinib, and paclitaxel compared to those with high risk, suggesting that low risk was indicative of increased sensitivity to the above drugs (**Figures 5A–H**). Therefore, the NRlncRNA signature could act as a potential chemotherapy predictor.

### Mutation Analysis and Tumor Mutation Burden Calculation

**Figure 6:**
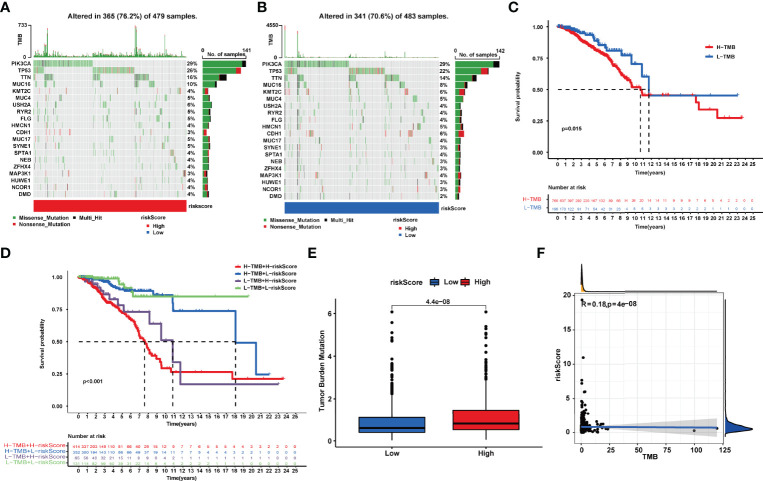
**(A, B)**. Tumor somatic mutation waterfall chart established from patients with high and low riskScores: **(A)** High-riskScore group and **(B)** low-riskScore group. **(C)** Kaplan–Meier survival analyses of TMB in BRCA patients on overall survival. **(D)** Kaplan–Meier survival analyses of TMB and riskScore on overall survival. **(E)** The relationship between TMB and riskScore groups. **(F)** Correlation analysis of the riskScore and tumor mutation load.

We observed a broader TMB in the high-risk group with 365 (76.2%) of 479 patients compared to the low-risk group. PIK3CA, TP53, and TTN had the most genetic alteration, of which mutation frequency was all over 10%. The results revealed a potential interaction between individual somatic mutations and riskScore (**Figures 6A, B**). The K-M curves showed a significantly better OS in the low-TMB group compared with the high-TMB group (**Figure 6C**). Moreover, we noticed that the tumor mutation load (TMB) was closely related to the riskScore with *R* = 0.18, *p* < 4e-08 (**Figure 6F**); the higher the riskScore, the higher the TMB (**Figure 6E**). Then, the two factors were taken into account together; the patients with a low riskScore and low TMB had the best prognosis; meanwhile, the patients with a high riskScore and high TMB had the worst prognosis (**Figure 6D**).

### Gene Set Enrichment Analysis and Gene Set Variation Analysis Between the High- and Low-Risk Groups

**Figure 7:**
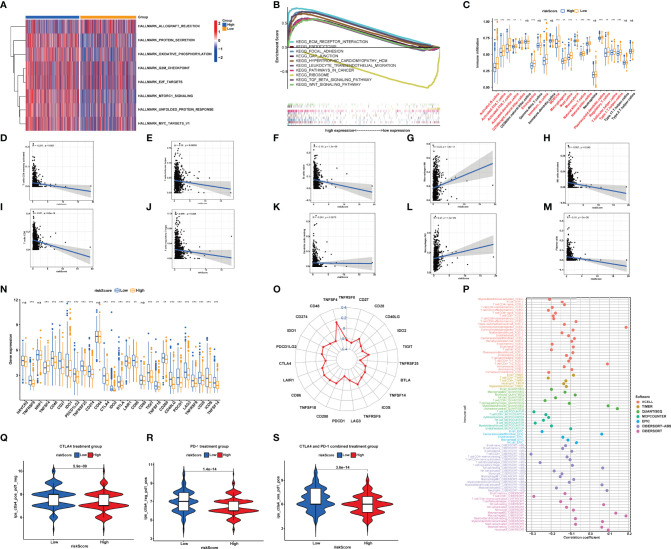
**(A)** Different pathways between the high-risk and low-risk groups. **(B)** Enrichment analyses of biological functions and pathways in the high- and low-risk group. **(C)** Single-sample gene set enrichment analysis of immune status between low- and high-risk subgroups. **(D–M)** Correlation between the distribution of tumor immune cells and value of NRlncRNA riskScore. **(N, O)** Comparisons of the expression levels of immune checkpoints between two groups. **(P)** The immune cell bubble of risk groups. **(Q–S)**. Treatment effects of CTLA-4 or PD-1 and combined CTLA-4 and PD-1 were evaluated in patients with high and low riskScores. **(Q)** CTLA-4 treatment group, **(R)** PD-1 treatment group, and **(S)** CTLA-4 and PD-1 combined treatment group. *P<0.05; **P<0.01; ***P<0.001. ns, P>0.05.

After GSEA was performed, we observed that several pathways with enrichment in the high-risk group were related to immunity with the filter criteria of FDR *q*-value<0.05 (**Table S4**), including “ECM_RECEPTOR_INTERACTION”, “FOCAL_ADHESION”, and “GAP_JUNCTION” (**Figure 7A**). Based on the calculated enrichment score of each sample, we identified enriched-pathway variation between the low-risk and high-risk group using the GSVA method (FDR < 0.05). We observed that from the low-risk to the high-risk group, the enrichment score was obviously increased in “HALLMARK_PROTEIN_SECRETION”, “MTORC1_SIGNALING”, “MYC_TARGETS_V1”, “OXIDATIVE_PHOSPHORYLATION”, “UNFOLDED_PROTEIN_RESPONSE”,”G2M_CHECKPOINT”, and “E2F_TARGETS”. The above results indicating the NRlncRNAs may affect immune-related mechanisms (**Figure 7B**).

### Potential Application of Necroptosis LncRNA Signature for Predicting Tumor Immune Microenvironment and Immunotherapy Responses

Subsequently, we used the “CIBERSORT” algorithm to investigate the correlation between riskScore and tumor-infiltrating immune cell (TIC) infiltration (**Table S5**). The relative proportion of immune cells in the BRCA samples by the “CIBERSORT” algorithm can be seen in **Figure S1**. The scatter plots showed the association between riskScore and the proportion of related TIC species (*p* < 0.05) in BRCA samples. We observed that the value of riskScore was positively correlated with the infiltrating levels of M2 macrophages and M0 macrophages, while the value of riskScore was negatively associated with the infiltrating levels of naive B cell, resting dendritic cells, activated NK cells, plasma cells, CD4+ T cells, CD8+ T cells, CD4+ memory T cells, follicular helper CD4 T cells, and regulatory T cells (**Figures 7D–M**).

We furthermore aimed to explore the relationship between the risk groups and immune cell infiltration by calculating the number of immune cells in BRCA using ssGSEA (**Table S6** and **Figure 7C**). The results demonstrated that the contents of CD56+ NK cells, γδ-T cells, immature dendritic cells, neutrophils, and type 17 T-helper cells did not show a significant difference among the 28 types of immune cells between two groups. The other 22 types of immune cells were decreased in the high-risk group (**Figure 7C**). Thus, it was concluded that low risk is a type of immune activation, while high risk is a type of immune failure. The above results were validated based on XCELL, CIBERSORT-ABS, TIMER, QUANTISEQ, MCPCOUNTER, EPIC, and CIBERSORT-ABS algorithms (**Figure 7P**). According to the conclusion, we speculated that the riskScore may be significantly correlated with regulating immunity and then affecting the prognosis of BRCA patients.

Synthesizing the results of ssGSEA and CIBERSORT, we came to a conclusion: the riskScore was negatively correlated with the infiltrating levels of naive B cells, resting dendritic cells, activated NK cells, plasma cells, CD4+ memory T cells, CD8+ T cells, follicular helper T cells, and regulatory T cells, indicating that the riskScore may affect tumor-infiltrating immune cell (TIC) infiltration.

### Immune Checkpoint and Immunotherapy

Immune checkpoint inhibitors (ICIs) were a rising and valid treatment strategy targeting numerous species of cancers; filtrating patients sensitive to ICIs will be beneficial to precise and effective medicine. As previous results showed that riskScore was a dependable prognostic factor, and was associated with TMB and tumor infiltration, we then aimed to verify the ability of riskScores in predicting immunotherapeutic benefits. We observed that a lower riskScore indicated a higher expression level of the other immune checkpoints except for TNFSF4 (**Figures 7N, O**).

In TCIA, the IPS (immunophenoscore), which was based on immunogenicity, could achieve a high accuracy on predicting the immunotherapy response of patients. Therefore, we analyzed the relationship of IPS between high- and low-riskScore groups. We perceived that in the CTLA-4 and PD-1 groups, patients in the low-riskScore group both showed better treatment effects (CTLA-4: 5.9e−09; PD-1: 1.4e-14) (**Figures 7Q, R**). In the CTLA-4 and PD-1 combined treatment group, patients in the high-m6A score group still indicated better treatment effects (*p* = 3.6e−14) (**Figure 7S**), which meant that patients accepting the treatment of both PD-L1 and CTLA4 showed superior reactivity of immune response. The result provided us advice in clinical practice on whether to use and what to use for immunotherapy. Overall, the riskScore established by us had great potential in predicting prognosis and immunotherapeutic benefits, which may provide sally ports for us to provide individualized and precise treatment.

### Identification of Necroptosis-Related Molecular Phenotypes

**Figure 8:**
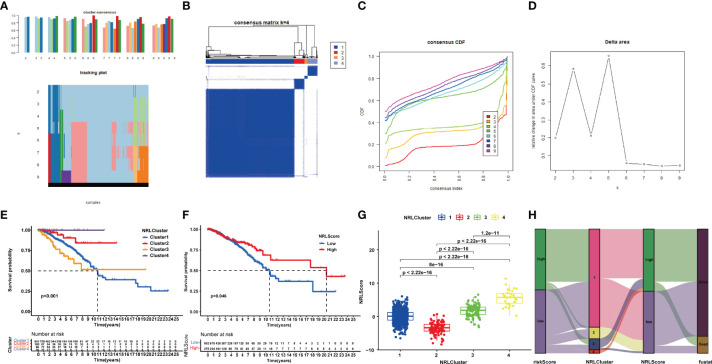
Consensus clustering of 22 NRlncRNAs identified four clusters of patients. **(A)** The tracking plot for *k* = 2 to *k* = 9. **(B)** The heatmap for *k* = 4. **(C)** Consensus clustering CDF with *k* = 2 to *k* = 9. **(D)** Relative change in area under the CDF curve for *k* = 2–9. **(E)** KM curve of the survival difference among clusters 1–4. **(F)** KM curve of the survival difference between high- and low-risk groups. **(G)** Correlation between NRLCusters and NRLScore. **(H)** Sankey diagram showing the co-expression of riskScores, NRLClusters, and NRLScores.

Based on the expression profiles of the 22 NRlncRNAs in the signature, we performed consensus clustering. *k* = 4 was identified with optimal clustering stability from *k* = 2 to 9, which showed the greatest correlation within the group and a low correlation among groups (**Figure 8B**), suggesting the practicability of dividing the patients into four clusters based on 22 NRlncRNAs. A consensus cumulative distribution function (CDF) diagram showed that when *k* = 4, CDF reached an approximate maximum (**Figure 8C**), and classification was robust (**Figures 8A, D**). The K-M curve revealed that patients in NRLCluster 4 had the best OS, while those in NRLCluster 3 had the worst OS (**Figure 8E**).

### Development of NRLScore to Quantify Individual Necroptosis Pattern

In view of the individual heterogeneity and complexity of BRCA patients, we calculated NRLScore based on the PCA on the 22 NRlncRNAs in the model. We defined NRLScore = PC1+PC2 to quantify the individual necroptosis pattern of BRCA patients and further to facilitate precise treatment. As indicated from the K-M curve, patients with a higher NRLScore had a better OS (**Figure 8F**). We also observed that NRLScores of patients in ferrCluster 4 were significantly higher than NRLClusters 1, 2, and 3 (**Figure 8G**). The Sankey diagram shows the attribute changes in riskScore, NRLCluster, NRLScore, and survival status, indicating that the higher the riskScore and the lower the NRLScore, the higher the risk of death (**Figure 8H**). The above results enriched treatment strategies for BRCA patients not only in targeted therapy and chemotherapy but also in immunotherapy.

## Discussion

Immunotherapy is a rapidly evolving concept that has been given the tag “fifth pillar” of cancer therapy (33), which has shown clinical efficacy in a variety of cancers (34), and has become an established form of cancer treatment (35). Historically, BRCA has been considered to be an immunogenic “cold” tumor. However, the appearance of ICIs resulted in immunotherapy becoming an emerging new treatment modality for BRCA (36). BRCA subtypes are both genetically and phenotypically distinct, and response rates to immunotherapy in BRCA vary among the different clinical subtypes of BRCA, which may not be the optimal classification to assess immunotherapy sensitivity (37).

Necroptosis plays an integral part in the induction and amplification of cancer immunity (10). RIPK3 is required to regulate cytokine expression in DCs, which is a key sentinel in regulating immune homeostasis (38). As is reported, necroptosis occurs during the late stage of T-cell proliferation and necroptotic signaling is markedly intensified in T cells absent in FADD, suggesting that FADD may negatively regulate necroptosis mediated by T-cell receptors (39). Furthermore, necroptosis initiates adaptive immune responses by releasing DAMPs into the tissue microenvironment (40). The TME status is the leading cause of the differential responses and outcomes in cancer patients receiving the same treatment, especially for multiple immunotherapies (41, 42). Therefore, explaining the diversity and complexity of TME is an indispensable step to enhance the predictive power and clinical guidance of immunotherapy.

Extensive interest in cancer immunotherapy is reported according to the clinical importance of CTLA-4 and PD-1/PD-L1 [programmed death (PD) and programmed death-ligand (PD-L1)] in immune checkpoint therapies (43). The main immune checkpoints for BRCA include cytotoxic T-lymphocyte-associated protein-4 (CTLA-4), programmed death receptor 1/programmed cell death ligand 1 (PD-1/L1), lymphocyte activation gene 3 (LAG-3), T-cell immunoglobulin domain and mucin 3 (TIM-3), and other molecules (44). Clinical trials like SOLTI-1503 PROMETEO TRIAL (45), KEYNOTE-086 (46), NIMBUS (47), KEYNOTE-173 (48), KEYNOTE-522 (49), and KEYNOTE-355 (50) showed that ICIs have made significant progress in BRCA immunotherapy, which is expected to become a new treatment for BRCA.

In this study, 46 NRlncRNAs were obtained by using the univariate Cox regression analysis. To prevent model overfitting, we performed LASSO regression analysis to identify 22 key NRlncRNAs, and multivariate Cox regression analysis was applied to calculate coefficients and construct the risk model. The K-M curves showed that patients in the low-risk group had longer survival than those in the high-risk group. Afterwards, we established forest plots and ROC plots including age, sex, T-stage, N-stage, M-stage, AJCC stage, and risk scores. By plotting a risk heatmap, a risk curve, an ROC curve, and a survival curve, we drew a conclusion that the risk model indeed had a good predictive effect. Meanwhile, we obtained similar results in the test cohort. According to the results of GSEA and GSVA, it was concluded that these two groups were associated with immunity. Then, ssGSEA and CIBERSORT algorithms were used to assess the status of the immune cell infiltration of each patient, and we found out that the low-risk group could be described as the immune-activated type, while the high-risk group could be described as the immune failure type. Meanwhile, the riskScore has a positive correlation with TMB; the higher the TMB, the worse the prognosis. The PD-L1 combined CTLA4 immunotherapy seemed suitable for patients who had a lower riskScore. Finally, we identified three necroptosis-related molecular patterns using consensus clustering analysis.

Compared with existing signature makers, Xu et al. provided an RNA binding protein-related lncRNA prognostic signature for prognosis (51), Yan et al. built a signature for CRISPR-Cas9-Based Cancer Dependency Map Genes (52), and Zou et al. identified glycolysis-related lncRNAs (53); the NRlncRNA signature showed higher values of AUCs and performed better prediction of prognosis in stratified risk analysis of survival. However, we also noticed TNBC patients with a lower riskScore, which seemed not so rational. We thought that based on our small sample size (we chose the BRCA patients whose clinicopathological parameters were complete), a certain degree of deviation rather than the NRlncRNAs signature itself might contribute to this strange phenomenon. Nonetheless, there are certain limitations to our study. First, our conclusions were only based on the datasets from TCGA. In other words, only retrospective datasets were used to identify our conclusion. Thus, a large, prospective, and multicenter clinical cohort is needed to confirm and improve the accuracy of the model. Moreover, the range of studies included all subtypes of BRCA. However, anti-PD-1/PD-L1 or anti-CTLA4 was mainly used in triple-negative breast cancer (TNBC) (54–56); thus, we would choose TNBC patients in further studies. Finally, the specific mechanism of necroptosis-related lncRNAs in BRCA and their interconnection with immunity are not yet fully understood; we will verify the expression levels of LINC00377, MEF2C-AS1, LMNTD2-AS1, and LINC02446 in patients in Zhejiang Provincial People’s Hospital rather than only in BRCA cells, and more experimental studies are needed to reveal the detailed molecular mechanisms in BRCA of the NRlncRNAs in the signature.

## Conclusions

Our research constructed a novel NRlncRNA signature that is useful for predicting the survival outcome of patients with BRCA to evaluate the TME immune cell infiltration characteristics of a single patient with BRCA. Furthermore, it also showed superior predictive power in clinical response to immunotherapy. In short, our results provide insights to improve personalized cancer immunotherapy and to distinguish the drug response of patients with BRCA well.

## Data Availability Statement

The original contributions presented in the study are included in the article/**Supplementary Material**. Further inquiries can be directed to the corresponding authors.

## Author Contributions

XM and QX substantially contributed to the conception of the work. YX, TZ, and BY contributed to the data collection. YX and QZ performed the nomogram model analyses and wrote the manuscript. YX, BY, and TZ helped to perform the enrichment and network analysis. YX, QZ, XM and QX drafted and revised the manuscript. All authors contributed to the article and approved the submitted version.

## Funding

This research was supported by grants from the National Natural Science Foundation of China (81973861) and the Zhejiang Provincial Ministry Medical and Health Co-construction Major Project (20214355173).

## Conflict of Interest

The authors declare that the research was conducted in the absence of any commercial or financial relationships that could be construed as a potential conflict of interest.

## Publisher’s Note

All claims expressed in this article are solely those of the authors and do not necessarily represent those of their affiliated organizations, or those of the publisher, the editors and the reviewers. Any product that may be evaluated in this article, or claim that may be made by its manufacturer, is not guaranteed or endorsed by the publisher.

## Supplementary Material

Supplementary Figure 1 The relative proportion of immune cells in the BRCA samples.

Supplementary Table 1 1520 necroptosis-related lncRNAs (NRlncRNAs) (correlation coefficients >0.4 and *p* < 0.001).

Supplementary Table 2 46 prognostic NRlncRNAs (p < 0.05).

Supplementary Table 3 riskScores of patients in train and test cohorts.

Supplementary Table 4 Gene set enrichment analysis between the high-risk and low-risk groups.

Supplementary Table 5 “CIBERSORT” algorithm to investigate the correlation between riskScore and tumor-infiltrating immune cells (TIC) infiltration.

Supplementary Table 6 The relationship between the risk groups and immune cells infiltration *via* calculating the number of immune cells in BRCA using single-sample gene set enrichment analysis.

## Abbreviations

TCGA, The Cancer Genome Atlas; Lasso, least absolute shrinkage and selection operator; TME, tumor microenvironment; UCSC, University of California, Santa Cruz; TCIA, The Cancer Immunome Atlas; IC_50_, half-maximum inhibitory concentration; ROC, receiver operating characteristic; NRG, necroptosis-related gene; NRlncRNA, necroptosis-related lncRNA; NRCluster, necroptosis-related cluster; NRLScore, necroptosis-related lncRNA score; CNV, copy number variation; GSVA, the gene set variation analysis; ssGSEA, single-sample gene set enrichment analysis; TMB, tumor mutation burden; PCA, principal component analysis; ICI, immune checkpoint inhibitor; TIC, tumor-infiltrating immune cell; IPS, immunophenoscore.
