# Supplementary figures and images for: Necroptosis-Related LncRNAs Signature and Subtypes for Predicting Prognosis and Revealing the Immune Microenvironment in Breast Cancer
Source: Front Oncol. 2022 May 24;12:887318. doi: 10.3389/fonc.2022.887318 (PMC9171493; doi:10.3389/fonc.2022.887318)

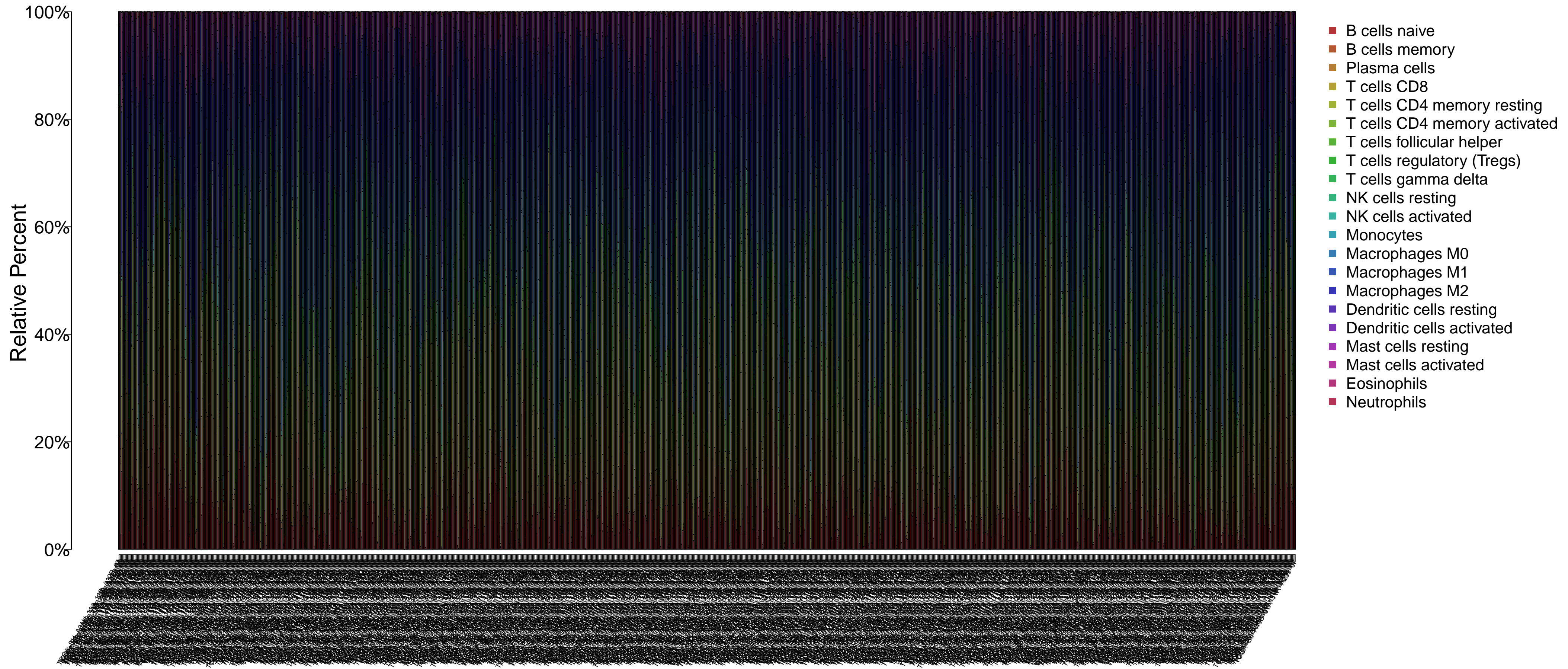

Supplement: Supplementary Figure 1 — The relative proportion of immune cells in the BRCA samples. [file DataSheet_1.pdf]
